# Adjustment for energy intake in nutritional research: a causal inference perspective

**DOI:** 10.1093/ajcn/nqab266

**Published:** 2021-07-27

**Authors:** Georgia D Tomova, Kellyn F Arnold, Mark S Gilthorpe, Peter W G Tennant

**Affiliations:** Leeds Institute for Data Analytics, University of Leeds, Leeds, United Kingdom; Faculty of Medicine and Health, University of Leeds, Leeds, United Kingdom; Alan Turing Institute, London, United Kingdom; Leeds Institute for Data Analytics, University of Leeds, Leeds, United Kingdom; Faculty of Environment, University of Leeds, Leeds, United Kingdom; Leeds Institute for Data Analytics, University of Leeds, Leeds, United Kingdom; Faculty of Medicine and Health, University of Leeds, Leeds, United Kingdom; Alan Turing Institute, London, United Kingdom; Leeds Institute for Data Analytics, University of Leeds, Leeds, United Kingdom; Faculty of Medicine and Health, University of Leeds, Leeds, United Kingdom; Alan Turing Institute, London, United Kingdom

**Keywords:** nutritional epidemiology, estimand, causal inference, compositional data, directed acyclic graphs

## Abstract

**Background:**

Four models are commonly used to adjust for energy intake when estimating the causal effect of a dietary component on an outcome: *1*) the “standard model” adjusts for total energy intake, *2*) the “energy partition model” adjusts for remaining energy intake, *3*) the “nutrient density model” rescales the exposure as a proportion of total energy, and *4*) the “residual model” indirectly adjusts for total energy by using a residual. It remains underappreciated that each approach evaluates a different estimand and only partially accounts for confounding by common dietary causes.

**Objectives:**

We aimed to clarify the implied causal estimand and interpretation of each model and evaluate their performance in reducing dietary confounding.

**Methods:**

Semiparametric directed acyclic graphs and Monte Carlo simulations were used to identify the estimands and interpretations implied by each model and explore their performance in the absence or presence of dietary confounding.

**Results:**

The “standard model” and the mathematically identical “residual model” estimate the average relative causal effect (i.e., a “substitution” effect) but provide biased estimates even in the absence of confounding. The “energy partition model” estimates the total causal effect but only provides unbiased estimates in the absence of confounding or when all other nutrients have equal effects on the outcome. The “nutrient density model” has an obscure interpretation but attempts to estimate the average relative causal effect rescaled as a proportion of total energy. Accurate estimates of both the total and average relative causal effects may instead be derived by simultaneously adjusting for all dietary components, an approach we term the “all-components model.”

**Conclusions:**

Lack of awareness of the estimand differences and accuracy of the 4 modeling approaches may explain some of the apparent heterogeneity among existing nutritional studies. This raises serious questions regarding the validity of meta-analyses where different estimands have been inappropriately pooled.

See corresponding editorial on page 3.

## Introduction

Estimating the causal effect of an individual dietary component on ≥1 health outcomes is a common practice in nutrition research. The purported aim is to identify foods or nutrients that are particularly beneficial or harmful to health, and hence reveal potential targets for public health or policy intervention. For example, many countries have introduced taxes on sugar-sweetened beverages because of their high concentration of nonmilk extrinsic sugars and their estimated contribution to the risks of obesity and other adverse health outcomes (1, 2).

Randomized controlled trials are generally difficult to perform in larger samples over longer time periods, and the effects observed may not generalize to dietary practices in the target population (3). Nutrition research is therefore highly reliant on the analysis of observational data, which brings several challenges for causal inference. One of the biggest of these is how to separate the effects of individual dietary components from the effects of the overall diet. Typically, those who consume a greater quantity of any 1 dietary component will also consume a greater overall quantity of food and have a greater overall energy intake (4). Those who consume a greater overall quantity of food are often also systematically different in several other important ways, such as body size and composition (4). Separating the effect of a single nutrient exposure from the effects of body size, body composition, metabolic efficiency, and overall energy intake is, however, extremely challenging (5). Identifying the exact physiological, psychological, and sociocultural determinants of dietary intake and composition is not straightforward. Many of these determinants cannot be measured directly or are simply unknown. Overall energy intake is often the best available “proxy” for these determinants and is hence routinely used to address confounding (4). A proxy represents an observed variable that captures some or all of the variation of another unobserved variable.

Several strategies have been proposed to “control” (i.e., statistically adjust) for differences in overall energy intake when attempting to estimate the effects of individual dietary components, and there has been considerable debate about which of these strategies is most appropriate (4–9). The 2 most common approaches are the “standard model,” which involves adjusting for total energy (i.e., total intake of calories from all sources including the nutrient exposure of interest); and the “energy partition model,” which involves adjusting for the remaining energy intake (i.e., the intake of calories from all sources excluding the exposure nutrient of interest). A third approach, known as the “nutrient density model,” involves examining the nutrient exposure as a proportion (percentage) of total energy, with or without further adjustment for total energy. Finally, the “residual model” involves adjusting for the residual produced by regressing the nutrient exposure on total energy.

Although ostensibly similar in purpose, the choice of energy adjustment strategies has important implications, for both the causal effect being targeted (i.e., the estimand) and the accuracy of the estimate obtained (8). In theory, applied researchers are encouraged to select the adjustment strategy that is most compatible with their research question, or otherwise to carefully present and interpret different estimates (8). However, in practice, there is often limited explicit justification given for the approaches adopted and reported. Even when the models are correctly interpreted, there is little or no explanation regarding which approach is most suitable, and instead several approaches are commonly used and compared, even when they target different causal effect estimands (10, 11).

In this study, we used directed acyclic graphs (DAGs) and simulations to clarify the estimand and appropriate causal interpretation for each adjustment strategy. We also explored the estimation performance of each strategy and their effectiveness at reducing confounding by common causes of dietary intake and composition. Throughout, we consider the illustrative example of the effect of nonmilk extrinsic sugars (referred to as “sugars” for simplicity) on fasting plasma glucose concentrations.

### Considering compositional nutrition data using DAGs

A key challenge in the analysis of nutritional data is recognizing that total energy, together with energy intake from individual dietary components, represents an example of compositional data (12). Data are compositional when a “whole” variable (i.e., total energy) can be divided into meaningful “part” variables which together sum to that whole (i.e., energy intake from individual dietary components) (13). Although we might consider these whole and part variables to be distinct, in reality they represent the same variable at different levels of aggregation.

**FIGURE 1:**
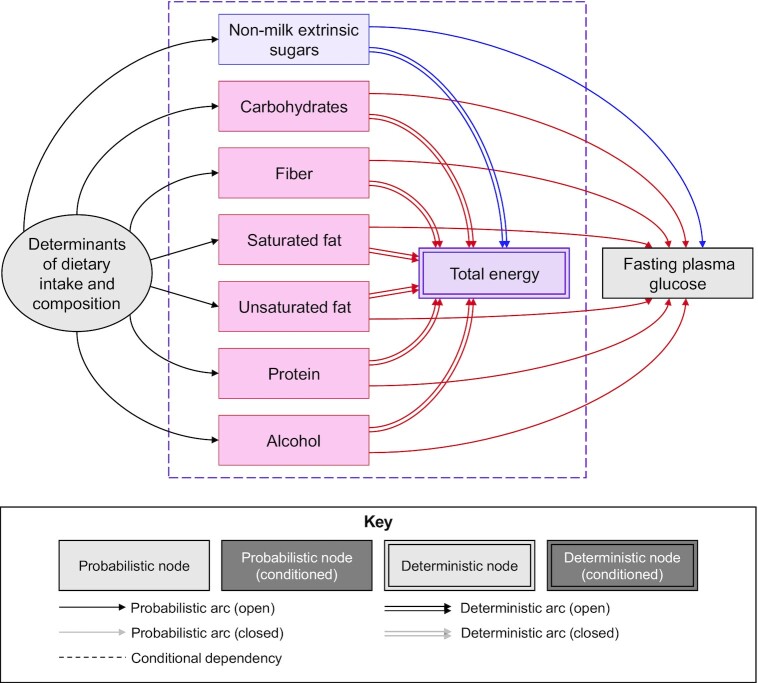
Directed acyclic graph showing the compositional nature of nutritional data. TE (the “whole,” purple) is fully determined by energy intake from 7 constituent macronutrients (the “parts,” blue and red). The nutrient exposure (nonmilk extrinsic sugars, blue) and 6 competing energy sources (red) all cause the outcome (GLUC concentration, gray) and are themselves caused by all unobserved determinants of dietary intake and composition (gray). The absence of an arc from energy intake to GLUC reflects the lack of an independent causal effect, conditional on all components of energy intake, i.e., any observed effect is the average combined causal effect of all nutrients on GLUC concentration.

Compositional data can be depicted using a type of semiparametric DAG, as introduced by Arnold et al. (14). DAGs are causal diagrams in which variables (nodes) are connected by unidirectional arrows (arcs) to depict hypothesized causal relations between them; no node may indirectly cause itself (15). Although they are generally used to depict probabilistic relations, they can also be used to depict situations for which the value of 1 variable is completely determined by ≥1 parent variables (16). We depict probabilistic and deterministic variables with single- and double-outlined rectangles, respectively, and probabilistic and deterministic relations with single- and double-lined arrows, respectively. We also place a dashed box around the compositional variables to highlight that they occur at the same point in time (14). The DAG in **Figure 1** depicts our illustrative scenario, where the energy intake from sugars and all other energy sources completely determines total energy and (probabilistically) affects fasting plasma glucose concentrations.

Depicting the relation in this way—with total energy as the “consequence” of the energy intake from all individual sources—is helpful for understanding the impact of different adjustment strategies because total energy can be conceptualized as a “collider.” A collider is a variable that is simultaneously caused by ≥2 other variables. Where the collider is completely determined by its individual components, as in the case of total energy, conditioning on the collider (i.e., holding its value fixed) creates a dependency between all the constituent components, such that any change in the value of 1 component must be accompanied by an equal but opposite average change in all other (unconditioned) components. Analyses that adjust for total energy therefore evaluate the “substitution” effect of exchanging the exposure with ≥1 other component nutrients (14). The compositional nature of the data should be recognized when selecting the most appropriate adjustment strategy.

### Total compared with relative causal effect estimands

Several authors have examined the interpretation of the different approaches to energy adjustment (4, 6–8), but none have explicitly considered the target estimand of each approach. This is likely because none of the models were developed within a formal causal framework. The estimand and estimating performance of each approach must therefore be inferred from theory.

**FIGURE 2:**
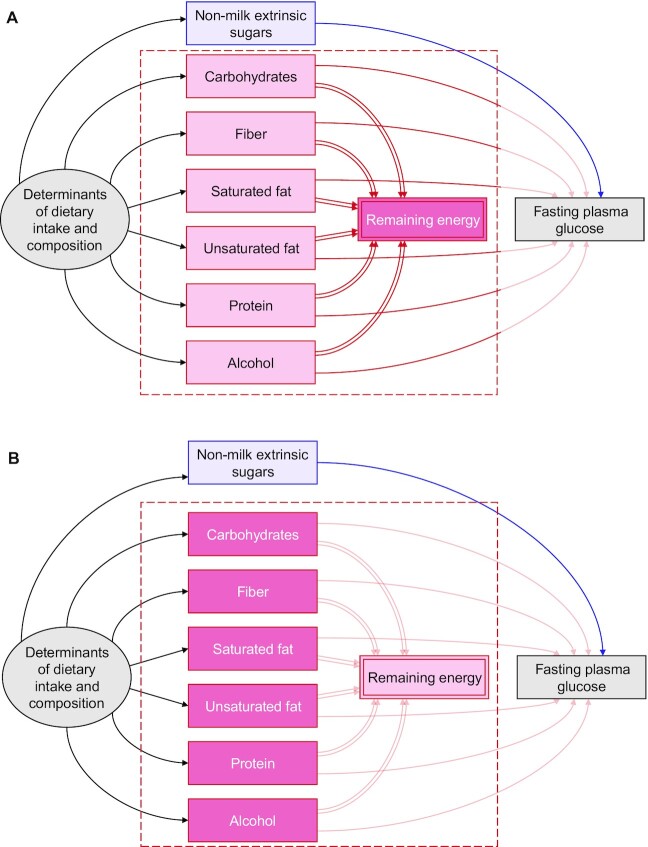
Directed acyclic graphs showing how confounding by common determinants of dietary intake and composition can be reduced when estimating the total causal effect (blue arc) of a nutritional exposure (e.g., nonmilk extrinsic sugars, blue) on an outcome (e.g., fasting plasma glucoseconcentration). Confounding by common determinants of dietary intake and composition will exist if ≥1 of the competing nutritional components (red) also cause the outcome (red arcs). This can be reduced (A) by conditioning on the remaining energy intake or (B) by conditioning on each of the competing nutritional components directly. For key see Figure 1.

The total causal effect of a nutrient exposure (e.g., sugars) on a health outcome (e.g., fasting plasma glucose) is the individual effect of increasing energy intake from that exposure while keeping all other sources of energy intake constant; this has previously been described as an “additive” effect (8). Because total energy is a “collider” between the exposure nutrient and the outcome, it should be evident that an unbiased estimate of this effect cannot be obtained by adjusting for total energy. Therefore, of the 4 most common adjustment approaches, only the energy partition model targets this effect. This method can be expected to improve the precision of the estimate (by reducing unexplained heterogeneity in the outcome) and reduce confounding bias from common causes of diet (**Figure 2**A). However, because the remaining energy intake is an average of all remaining sources of energy, some residual confounding can be expected wherever the remaining dietary components have distinct effects on the outcome. In this instance, adjusting simultaneously for all remaining dietary components—an approach that we term the “all-components model”—can be expected to provide a less biased estimate of the total causal effect than would be obtained by adjusting for the average remaining energy intake (Figure 2B).

**FIGURE 3:**
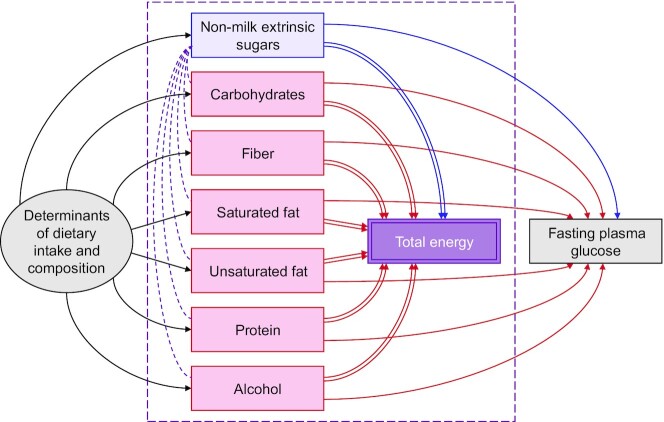
Directed acyclic graphs showing the consequences of adjusting for total energy when estimating the causal effect of a nutrient exposure (e.g., nonmilk extrinsic sugars, blue) on an outcome (e.g., GLUC concentration, gray). Total energy is completely determined by the exposure nutrient (nonmilk extrinsic sugars, blue) and all competing energy sources (red). Adjusting for TE (purple) opens conditional dependencies between the exposure and all competing energy sources (purple dashed arcs), so that the total causal effect (blue arc) is now in competition with the (average) effect of all competing energy sources (red arcs). The average relative causal effect thus represents the difference between the total causal effect of the exposure and the weighted average effect of all other energy sources. For key, see Figure 1.

A relative causal effect of a nutrient exposure is the joint effect of increasing energy intake from that nutrient while decreasing energy intake from ≥1 other energy sources to keep the total energy constant. In theory, there are many different relative causal effects that might be considered, requiring many different adjustment strategies. The average relative causal effect (also known as the weighted average compositional effect) (17) is the effect of a nutrient exposure relative to the weighted average effect of all other sources of energy, and is commonly estimated by adjusting for total energy (**Figure 3**). Other more specific average relative causal effects can also be targeted by in addition adjusting for specific competing sources of energy intake to remove them from the substitution group. However, as with remaining energy intake, adjusting for total energy is susceptible to residual confounding by common causes of diet if each residual dietary component has a distinct effect on the outcome. Even in the absence of confounding, models that rely on adjusting for summary variables—such as total energy—will likely experience bias, because it is rare for the effect of a summary or composite variable to match the weighted average effect of all the components therein. This phenomenon—which stems from the loss of information when ≥2 variables are combined—may be considered a form of “composite variable bias” and is distinct from residual confounding. Once again, a less biased estimate of the average relative causal effect may be derived from the “all-components model” (Figure 2B), by subtracting the true weighted average effect of all the residual sources of energy intake from the total causal effect of the exposure.

## Methods

### Illustrative example

To illustrate these principles, we explore the target estimand and the performance in estimating these quantities of the 4 standard energy adjustment strategies, the “all-components model,” and a reference unadjusted model, using simulated data. We consider the effect of sugar on fasting plasma glucose for a simple scenario in which the concentration of fasting plasma glucose is caused by the intake of 7 macronutrients (including sugar), either in the presence or absence of confounding by common causes of dietary intake and composition. In the absence of such confounding, any difference between the target estimand and the estimate obtained will represent composite variable bias. In the presence of confounding, any difference will be a combination of composite variable bias and residual confounding bias. We do not consider confounding that acts directly on the outcome (i.e., is independent of the remaining diet), because this is unaffected by the energy intake strategy.

### Data simulation

Standardized data were simulated using the “dagitty” (0.2-3) R package (18) to reflect the data generating process depicted in **Supplemental Figure 1**, where total energy was fully determined by the energy intake from 7 macronutrients: *1*) sugars, *2*) carbohydrates, *3*) fiber, *4*) saturated fat, *5*) unsaturated fat, *6*) protein, and *7*) alcohol. Total energy and remaining energy intake were not directly simulated. Instead, they were calculated from the sum of all macronutrient energy variables, or the sum of all energy variables except sugar, respectively. Each macronutrient was assigned a unique effect on fasting plasma glucose. Specific path coefficient values were chosen to represent plausible causal effects, and simulated variables were rescaled with plausible mean ± SD values informed by the National Diet and Nutrition Survey (see **Supplemental Table 1**) (19). All simulations and models (see below) were repeated in the presence of a single variable (U) that affects the intake of all macronutrients, to demonstrate the influence of confounding by common causes of dietary composition. Each simulation included 1000 observations and was repeated over 100,000 iterations. We report the median effect estimate and 2.5th and 97.5th centiles [representing the 95% simulation interval (SI)] from the 100,000 iterations for each model. For ease of illustration, effect estimates are expressed in mg/dL per 100 calories (mg/dL/100 kcal).

### Simulated effects

The total causal effect is the effect of increasing energy intake from the exposure of interest (i.e., sugars) while maintaining the same amounts of energy intake from all other sources. We simulated a total causal effect of 5.0 mg/dL/100 kcal, meaning that fasting plasma glucose increased by an average of 5.0 mg/dL for each additional 100 kcal of sugars consumed.

The average relative causal effect is the effect of increasing the energy intake from the exposure (i.e., sugars) while decreasing the energy intake from all other macronutrients to maintain the same total energy. We simulated an average relative causal effect of 2.0 mg/dL/100 kcal (equivalent to 0.4 mg/dL per 1% of total energy), meaning that fasting plasma glucose increased by an average of 2.0 mg/dL for each additional 100 kcal (or 0.4 mg/dL for each additional 1% of total energy) derived from sugars rather than from other macronutrient sources.

For simplicity, we assume that each macronutrient component has a linear and additive effect on the outcome and that the effect of each component is independent of all others.

### Models examined

We present and discuss the results obtained from using the following 6 models.

#### The unadjusted model (model 0)

(1) \begin{eqnarray*} \widehat {{\rm{GLUC}}} = \widehat {{a_0}}{\rm{\ }} + \widehat {{{\boldsymbol{a}}_1}}{\rm{NMES}} \end{eqnarray*}

This model targets the total causal effect of sugars (${\rm{NMES}}$) on fasting plasma glucose (${\rm{GLUC}}$). Because the model does not adjust for energy intake or any other variables, the coefficient $\widehat {{a_1}}$ should produce an unbiased estimate of the desired estimand where there is no confounding by common causes of diet.

#### The energy partition model (model 1)

(2) \begin{eqnarray*} \widehat {{\rm{GLUC}}} = \widehat {{b_0}}{\rm{\ }} + \widehat {{{\boldsymbol{b}}_1}}{\rm{NMES}} + \widehat {{b_2}}{\rm{RE}} \end{eqnarray*}

This model also targets the total causal effect of sugars (${\rm{NMES}}$) on fasting plasma glucose (${\rm{GLUC}}$) and attempts to minimize confounding by common causes of diet by adjusting for remaining energy intake (${\rm{RE}}$) (i.e., total intake of calories from all sources excluding sugars). Where there exists such confounding, the coefficient $\widehat {{b_1}}$ is therefore expected to produce a less biased estimate of the desired estimand than in the unadjusted model ($\widehat {{a_1}}$ in model 0); however, residual confounding may remain where competing nutrients have different effects on the outcome.

#### The standard model (model 2)

(3) \begin{eqnarray*} \widehat {{\rm{GLUC}}} = \widehat {{c_0}}{\rm{\ }} + \widehat {{{\boldsymbol{c}}_1}}{\rm{NMES}} + \widehat {{c_2}}{\rm{TE}} \end{eqnarray*}

This model targets the average relative causal effect of sugars (${\rm{NMES}}$) on fasting plasma glucose (${\rm{GLUC}}$) and attempts to minimize confounding by common causes of diet by adjusting for total energy (i.e., total intake of calories from all sources including sugars). In the absence of confounding, the coefficient $\widehat {{c_1}}$ is expected to provide a slightly biased estimate of this effect due to composite variable bias. This estimate is expected to experience further residual confounding bias in the presence of confounding where the competing nutrients have different effects on the outcome.

#### The nutrient density model (models 3a and 3b)

(4) \begin{eqnarray*} \widehat {{\rm{GLUC}}} = \widehat {{d_0}}{\rm{\ }} + \widehat {{{\boldsymbol{d}}_1}}{\rm{\ }}\frac{{{\rm{NMES}}}}{{{\rm{TE}}}}\left( { + {\rm{\ }}\widehat {{d_2}}{\rm{TE}}} \right) \end{eqnarray*}

This model involves transforming the nutrient exposure into a proportion (percentage) of total energy; additional adjustment may also be made for ${\rm{TE}}$, an approach that has been termed the “multivariable nutrient density model” (4). The estimand targeted by this model is unclear, but it is plausibly an attempt to estimate the average relative causal effect of sugars (${\rm{NMES}}$) on fasting plasma glucose (${\rm{GLUC}}$), rescaled as a percentage of total energy (i.e., 0.4 mg/dL/1%). However, the coefficient $\widehat {{d_1}}$ represents an obscure quantity that conflates both the effect of the nutrient exposure and that of the reciprocal of total energy, and it is therefore expected to be misleading and experience composite variable bias regardless of confounding by common causes of diet. We present models with (3a) and without (3b) adjustment for ${\rm{TE}}$.

#### The residual model (model 4)

(5) \begin{eqnarray*} \widehat {{\rm{GLUC}}} = {\rm{\ }}\widehat {{e_0}} + \widehat {{{\boldsymbol{e}}_1}}{\rm{NMES^{\prime}}} \end{eqnarray*}

This 2-stage approach involves regressing the nutrient exposure on total energy ($\widehat {{\rm{NMES}}} = \ \widehat {{x_0}} + \widehat {{x_1}}{\rm{TE}})$, and entering the model residual $[{\rm{NME}}{{\rm{S}}^{\rm{^{\prime}}}} = {\rm{\ NMES}} - \widehat {({x_0}} + \widehat {{x_1}}{\rm{TE}})]\ $ into a second unadjusted model. The approach is mathematically identical to the standard model, and therefore also targets the average relative causal effect of sugars (${\rm{NMES}}$) on fasting plasma glucose (${\rm{GLUC}}$). As in the standard model, the coefficient $\widehat {{e_1}}$ is expected to provide a slightly biased estimate in the absence of confounding due to composite variable bias, with further residual confounding bias occurring in the presence of confounding where the competing nutrients have different effects on the outcome.

#### The all-components model (model 5)

(6) \begin{eqnarray*} \widehat {{\rm{GLUC}}} &=& \widehat {{f_0}}{\rm{\ }} + \widehat {{{\boldsymbol{f}}_1}}{\rm{NMES}} + \widehat {{f_2}}{\rm{CRB}} + \widehat {{f_3}}{\rm{FBR}} + \widehat {{f_4}}{\rm{SF}}\\ && + \widehat {{f_5}}{\rm{UF}} + \widehat {{f_6}}{\rm{PRO}} + {\rm{\ }}\widehat {{f_7}}{\rm{ALC}} \end{eqnarray*}

This model targets the total causal effect of sugars (${\rm{NMES}}$) on fasting plasma glucose (${\rm{GLUC}}$) by adjusting for all individual component sources of energy: carbohydrates (${\rm{CRB}}$), fiber (${\rm{FBR}}$), saturated fat (${\rm{SF}}$), unsaturated fat (${\rm{UF}}$), protein (${\rm{PR}}$), and alcohol (${\rm{ALC}}$). Because this approach does not involve the use of summary or composite variables, the coefficient estimate $\widehat {{f_1}}$ is expected to provide an unbiased estimate of this effect regardless of confounding by common causes of diet.

An unbiased estimate of the average relative causal effect (say, ${\hat{g}_1}$) can also be derived using this model. This is achieved by subtracting a weighted average of the estimated effects of all other individual component sources of energy from the total causal effect of the exposure (i.e., ${\hat{g}_1} = {\hat{f}_1}\ - [ {\mathop \sum \limits_2^n {w_i}{{\hat{f}}_i}} ]$, where ${w_i}$ is the proportion of the remaining energy intake contributed by each component $i\ = \ \{ {2,\ \ldots ,\ n} \}$).

## Results

**Table 1** gives full results from the 6 models considered.

**TABLE 1:** Regression coefficients (and 95% SIs) for the effect of sugars on fasting plasma glucose estimated using 4 different causal estimand scenarios and adjustment approaches^1^

Model number	Model	Model name	Estimand	True estimate	Model estimate (95% SI), no confounding	Model estimate (95% SI), with confounding
0	$\ \widehat {{\rm{GLUC}}} = \widehat {{a_0}}{\rm{\ }} + \widehat {{{\boldsymbol{a}}_1}}{\rm{NMES}}$	The unadjusted model	Total causal effect	5.00^2^	5.00^2^ (3.80, 6.21)	8.22^2^ (7.09, 9.35)
1	$\ \widehat {{\rm{GLUC}}} = \widehat {{b_0}}{\rm{\ }} + \widehat {{{\boldsymbol{b}}_1}}{\rm{NMES}} + \widehat {{b_2}}{\rm{RE}}$	The energy partition model	Total causal effect	5.00^2^	5.00^2^ (3.95, 6.06)	5.49^2^ (4.53, 6.45)
2	$\ \widehat {{\rm{GLUC}}} = \widehat {{c_0}}{\rm{\ }} + \widehat {{{\boldsymbol{c}}_1}}{\rm{NMES}} + \widehat {{c_2}}{\rm{TE}}$	The standard model	Average relative causal effect	2.00^2^	1.94^2^ (0.83, 3.04)	2.28^2^ (1.22, 3.34)
3a	$\ \widehat {{\rm{GLUC}}} = \widehat {{d_0}}\ + \widehat {{{\boldsymbol{d}}_1}}\ \frac{{{\rm{NMES}}}}{{{\rm{TE}}}}$	The nutrient density model	Obscure^3^	0.40^4^	0.14^4^ (0.11, 0.40)	0.47^4,5^ (0.16, 0.75)
3b	$\ \widehat {{\rm{GLUC}}} = \widehat {{d_0}}\ + \widehat {{{\boldsymbol{d}}_1}}\ \frac{{{\rm{NMES}}}}{{{\rm{TE}}}} + {\rm{\ }}\widehat {{d_2}}{\rm{TE}}$	The multivariable nutrient density model	Obscure^3^	0.40^4^	0.35^4^ (0.14, 0.56)	0.39^4,5^ (0.18, 0.58)
4	$\ \widehat {{\rm{GLUC}}} = \ \widehat {{e_0}} + \widehat {{{\boldsymbol{e}}_1}}{\rm{NMES^{\prime}}}$	The residual model	Average relative causal effect	2.00^2^	1.94^2^ (0.83, 3.04)	2.28^2^ (1.22, 3.34)
5	$\ \widehat {{\rm{GLUC}}} = \widehat {{f_0}}{\rm{\ }} + \widehat {{{\boldsymbol{f}}_1}}{\rm{NMES}} + \widehat {{f_2}}{\rm{CRB}} + \widehat {{f_3}}{\rm{FBR}} + \widehat {{f_4}}{\rm{SF}} + \widehat {{f_5}}{\rm{UF}} + \widehat {{f_6}}{\rm{PRO}} + {\rm{\ }}\widehat {{f_7}}{\rm{ALC}}$	The all-components model	Total causal effect (${f_1})$	5.00^2^	5.00^2^ (3.95, 6.05)	5.00^2^ (3.95, 6.05)
			Average relative causal effect (${g_1} = {f_1}\ - [ {\mathop \sum \limits_2^n {w_i}{f_i}} ]$)	2.00^2^	2.00^2^ (0.87, 3.13)	2.00^2^ (0.88, 3.11)

1 PRO, proteinSI, simulation interval.

2 Values are expressed as mg/dL/100 kcal.

3 The nutrient density model evaluates an obscure estimand, but it is conceptually closest to the average relative causal effect rescaled as a proportion of total energy.

4 Values are expressed as mg/dL/1%.

5 The confounded estimates are closer to the true estimates than expected by chance because of the direction of confounding.

### The unadjusted model (model 0)

With no confounding by common causes of diet, the unadjusted model returns an unbiased estimate of the total causal effect ($\widehat {{a_1}}$ = 5.0 mg/dL/100 kcal). However, because the unadjusted model does not account for any competing sources of energy intake, the unadjusted model returns a severely biased estimate ($\widehat {{a_1}}$ = 8.2 mg/dL/100 kcal; 95% SI: 7.1, 9.4 mg/dL/100 kcal) when there is confounding.

### The energy partition model (model 1)

With no confounding, the energy partition model returns an unbiased estimate of the total causal effect ($\widehat {{b_1}\ }$= 5.0 mg/dL/100 kcal). With confounding, the energy partition model returns a more accurate (but still biased) estimate (${b_1}$ = 5.5 mg/dL/100 kcal; 95 SI: 4.5, 6.5 mg/dL/100 kcal) than the unadjusted model.

### The standard model (model 2)

With no confounding, the standard model returns a slightly biased estimate of the average relative causal effect ($\widehat {{c_1}}$ = 1.9 mg/dL/100 kcal; 95% SI: 0.8, 3.1 mg/dL/100 kcal). With confounding, the standard model also returns a biased estimate of the average relative causal effect ($\widehat {{c_1}}$ = 2.3 mg/dL/100 kcal; 95% SI: 1.2, 3.4 mg/dL/100 kcal).

### The nutrient density model (models 3a and 3b)

Model 3a: with no confounding, the nutrient density model returns a severely biased estimate of the average relative causal effect ($\widehat {{d_1}}$ = 0.14 mg/dL/%; 95% SI: −0.11, 0.40 mg/dL/%). With confounding, the nutrient density model also returns a biased estimate of the average relative causal effect ($\widehat {{d_1}}$ = 0.47 mg/dL/%; 95% SI: 0.16, 0.76  mg/dL/%).

Model 3b: with no confounding, the multivariable nutrient density model returns a more accurate estimate than the (unadjusted) nutrient density model, but one which is still biased ($\widehat {{d_1}}$ = 0.35 mg/dL/%; 95% SI: 0.14, 0.56 mg/dL/%). With confounding, the multivariable nutrient density model also returns a more accurate estimate than the (unadjusted) nutrient density model, but the estimate is still biased ($\widehat {{d_1}}$ = 0.39 mg/dL/%; 95% SI: 0.18, 0.58 mg/dL/%).

### The residual model (model 4)

With no confounding, the residual model returns the same (biased) estimate of the average relative causal effect as the standard model ($\widehat {{e_1}}$ = 1.9 mg/dL/100 kcal; 95% SI: 0.8, 3.1 mg/dL/100 kcal). With confounding, the residual model also returns the same (biased) estimate of the average relative causal effect as the standard model ($\widehat {{e_1}}$ = 2.3 mg/dL/100 kcal; 95% SI: 1.2, 3.4 mg/dL/100 kcal).

### The all-components model (model 5)

With no confounding, the all-components model returns unbiased estimates of the total causal effect ($\widehat {{f_1}}$ = 5.0 mg/dL/100 kcal; 95% SI: 4.0, 6.1 mg/dL/100 kcal) and average relative causal effect ($\widehat {{g_1}}$ = 2.0 mg/dL/100 kcal; 95% SI: 0.9, 3.1 mg/dL/100 kcal). Because this model involves adjusting for all remaining component sources of energy separately, in the presence of confounding by common causes of diet it also returns unbiased estimates of the total causal effect ($\widehat {{f_1}}$ = 5.0 mg/dL/100 kcal; 95% SI: 4.0, 6.0 mg/dL/100 kcal) and average relative causal effect ($\widehat {{g_1}}$ = 2.0 mg/dL/100 kcal; 95% SI: 0.9, 3.1 mg/dL/100 kcal).

## Discussion

### Overview

This study used DAGs and simulations to explore the target estimands and performance of the 4 standard approaches to adjusting for energy intake in nutritional research, as well as a fifth model that involves adjusting for all individual energy components. We demonstrate that the 4 standard approaches evaluate different estimands with different interpretations, and that none of the 4 methods provide robust estimates in the presence of confounding by common causes of dietary intake and composition in our simulated example. In contrast, the “all-components” model offers an accurate means to estimate both the total causal effect and the average relative causal effect. The nutrient density model conflates the effects of the exposure and total energy and does not target a meaningful estimand, regardless of bias.

### Principal findings

Our simulations highlight 4 important considerations for the analysis and interpretation of nutritional data.

First, different modeling strategies target different estimands. The energy partition model is the only 1 of the 4 standard approaches that targets the total causal effect of the nutrient exposure on the outcome (i.e., the “additive” effect of the exposure on top of the existing diet). In contrast, the standard model and the mathematically equivalent residual model both target the average relative causal effect of the nutrient exposure (i.e., the effect of “substituting” the exposure with other calorific sources to maintain the same total energy). The nutrient density model targets an obscure estimand that conflates the effects of the nutrient exposure and the (inverse) effect of total energy; this is conceptually closest to the average relative causal effect, rescaled as a proportion of the total energy.

Second, we show that none of the 4 standard approaches provide robust estimates of their respective causal estimands in the presence of confounding by common causes of dietary intake and composition. There are different reasons for this which depend on the model used. For example, the standard model and the energy partition model only remove the average effect of energy intake. In the energy partition model, the variable “remaining energy” provides an imperfect average of the combined effects of all remaining nutrients (i.e., not equal to 3.0 mg/dL/100 kcal). This does not bias the estimate of sugars in the model, because sugars and remaining energy are competing exposures (i.e., they do not introduce confounding bias). However, the issue becomes apparent when total energy is adjusted for (i.e., the standard model), because sugars and remaining energy become dependent on each other. The imperfect average that remaining energy captures is therefore included in the joint effect that is estimated and leads to biased coefficients. Residual confounding will therefore remain wherever the effect varies between different component energy sources. This assumption is, however, fundamental to the conduct of nutritional research, because there would otherwise be no justification for estimating the causal effect of ≥1 individual nutrient exposures. The residual model is algebraically identical to the standard model (6), and as such suffers the same problems while offering no additional benefits. The nutrient density model involves evaluating a proportion of energy intake (i.e., a ratio) as the exposure, rather than the absolute amount of the nutrient exposure consumed. For a variable expressed as a ratio, the individual causal effects of the constituent components cannot be separated and interpreted on their own. Ratio variables like these have obscure interpretations because they conflate the effects of the numerator and denominator and are not robust to confounding bias (20, 21). Adjusting for total energy—as in the multivariable nutrient density model—offers considerably more accurate estimates of average relative causal effects by reducing confounding and reducing the distorting joint effects of the total energydenominator, but nevertheless remains biased.

Third, even in the absence of confounding, both the standard model and residual model were affected by composite variable bias due to the loss of information that occurs when ≥2 components with distinct causal effects are combined into a single “total” variable. The nutrient density model produced moderately to severely biased estimates depending on whether additional adjustment was made for total energy.

Finally, we show that a model that includes all individual dietary components (i.e., the all-components model) offers a robust means to estimate both the total causal effect and the average relative causal effect. The all-components model is a special case of the energy partition model, in which adjustment is made for all individual dietary components but estimates are calculated using the weighted coefficients of all nutrients. The specific weights assigned to each nutrient depend on the causal effect estimand of interest. In a similar way, robust causal effect estimates could also be obtained using a variant of the standard model (or residual model) that in addition adjusted for all but 1 remaining dietary component, an approach that is sometimes termed the “leave-one-out” approach to estimating a specific relative (i.e., substitution) causal effect (22). However, because the coefficients from this model would not represent total causal effects, we would recommend the all-components model as the more intuitive and transparent option and the least susceptible to misinterpretation.

### Implications

None of the most common approaches to adjusting for energy intake in nutritional research provide robust estimates of meaningful causal effects. For some models, this is true even with no confounding by common causes of diet, reflecting fundamental issues with these approaches. This has serious implications for the validity and interpretation of existing studies that have used these models.

It remains underappreciated that adjusting for total energyand adjusting for remaining energy intake evaluate very different causal estimands. In our simulation, the true total causal effect of sugars on fasting plasma glucose was 5.0 mg/dL/100 kcal, and the true average relative causal effect was 2.0 mg/dL/100 kcal. These 2 estimands relate to very different questions that require very different interpretations. If this distinction is not recognized, there is a high chance of misinterpretation and confusion. Unfortunately, meta-analyses of dietary exposures rarely separate studies based on their target estimand and/or modeling strategy (1), resulting in confusing summary estimates that are difficult—if not impossible—to interpret causally. The inappropriate synthesis of estimates from different estimands may therefore render many meta-analyses meaningless.

Residual confounding is also likely to contribute to the heterogeneity of estimates observed in the literature, given the inadequacy of adjusting for energy intake using any of the traditionally recommended approaches. More robust estimates can be obtained by adjusting simultaneously for all dietary components (as in the “all-components model”), but this is not common practice. However, this strategy does introduce a trade-off between minimizing bias (by including the largest number of components at the finest level of detail) and maximizing precision (by having to estimate many parameters, i.e., 1 for each additional dietary component). Creating and adjusting for latent dietary profiles may offer a more parsimonious and hence more efficient approach to adjusting for common dietary causes, but there is still likely to be some accuracy trade-off (23).

### Recommendations

Studies that seek to estimate the causal effect of ≥1 dietary components on ≥1 outcomes should clearly state their target estimands of interest and justify an adjustment strategy for estimating this effect.

Meta-analyses should only attempt to pool estimates for identical estimands, and extra care should be taken by reviewers to determine the implied estimand where this is not explicit. DAGs offer a simple means to identify the appropriate adjustment set for a particular estimand, and guidelines are now available on how best to report their use (24).

A single model that includes all individual components of the diet may provide the simplest and most accurate approach to estimating both the total causal effect and any relative causal effect of interest.

### Strengths and weaknesses

Although useful for demonstrating theoretical concepts, data simulations are oversimplifications of reality. The true causal effects of sugar intake, and of all other macronutrients, on fasting plasma glucose are likely to differ from what was simulated. The specific values were selected to most clearly illustrate the issues at hand, and no effects reported in this study should be interpreted in the nutrition domain. Fasting plasma glucose and all dietary variables were simulated to be multivariate normal, with no measurement error, and with no interactions among variables, which does not reflect reality. On average, measurement error can be expected to produce diluted effect estimates, but in any individual sample the effect may act in any direction. Where the effect of the exposure interacts with ≥1 other variables, it will not be possible to estimate a single causal effect for the whole population. For simplicity, we assumed that each macronutrient component had a linear and additive effect on the outcome and that the effect of each component was independent of all others. In practice, it is possible that some nutritional exposures may have nonlinear effects and/or the effects may depend on other elements of the diet. In such circumstances, none of the common approaches to energy adjustment would provide accurate causal effect estimates, and more complex parameterizations would be needed. To aid demonstration, we transformed all simulated variables to have plausible means and SDs based on observations in official data sources, although this has no substantive impact on the results derived. Although we reduced the SDs to minimize the occurrence of negative values, some negative and biologically implausible values were nevertheless simulated in some instances which, although nonsensical, did not affect the validity of the simulations or the interpretation of results. For ease of interpretation, we examined all macronutrient components in calorie units. In practice, many studies use alternative units of measurement, e.g., grams or servings, which may lead to further confusion in the estimand being estimated and/or exaggerate problems with composite variable bias.

When the aim is to reduce confounding, overall energy intake is adjusted as a proxy of unobserved determinants of dietary intake and composition. Therefore, in this study we only considered confounding introduced by common causes of diet. Minimizing confounding by common causes of diet does not eliminate the need for a carefully considered adjustment set. In the presence of standard confounding (i.e., variables that cause both the exposure and the outcome), adjusting for all dietary components would not be sufficient to eliminate all confounding bias. In such circumstances, when estimating the causal effect of a nutrient exposure on a health outcome, the complete adjustment set should be carefully selected and justified, ideally using DAGs (24). We did not consider time-varying confounding, which is common in practice but would require more sophisticated simulation and modeling approaches that are not directly relevant to the energy adjustment strategy.

### Conclusion

It is not fully appreciated that the most common approaches to adjusting for energy intake in nutritional research target different estimands with different interpretations. Moreover, none of these approaches offer complete adjustment for confounding from common causes of dietary intake and composition. These 2 issues together may explain a large portion of the heterogeneity in effect estimates between nutritional studies. The alternative single model that includes all individual components of the diet may provide the simplest and most accurate approach to estimating total causal effects and any desired relative causal effects.

## Supplementary Material

nqab266_Supplemental_File

## Data Availability

Data described in the article, code book, and analytic code will be made publicly and freely available without restriction at https://github.com/georgiatomova/adjustment-energy-intake.

## Notes

The authors reported no funding received for this study. GDT is grateful for PhD funding from Alan Turing Institute grant EP/N510129/1. KFA, MSG, and PWGT are all supported by Alan Turing Institute grant EP/N510129/1.

Supplemental Figure 1 and Supplemental Table 1 are available from the “Supplementary data” link in the online posting of the article and from the same link in the online table of contents at https://academic.oup.com/ajcn/.

## References

[1] Malik VS, Pan A, Willett WC, Hu FB. Sugar-sweetened beverages and weight gain in children and adults: a systematic review and meta-analysis. Am J Clin Nutr. 2013;98(4):1084–102. 23966427 10.3945/ajcn.113.058362 PMC3778861

[2] Cabrera Escobar MA, Veerman JL, Tollman SM, Bertram MY, Hofman KJ. Evidence that a tax on sugar sweetened beverages reduces the obesity rate: a meta-analysis. BMC Public Health. 2013;13:1072. 24225016 10.1186/1471-2458-13-1072 PMC3840583

[3] Weaver CM, Miller JW. Challenges in conducting clinical nutrition research. Nutr Rev. 2017;75(7):491–9. 28605476 10.1093/nutrit/nux026 PMC5654371

[4] Willett WC, Howe GR, Kushi LH. Adjustment for total energy intake in epidemiologic studies. Am J Clin Nutr. 1997;65(4):1220S–8S. 9094926 10.1093/ajcn/65.4.1220S

[5] Willett W, Stampfer MJ. Total energy intake: implications for epidemiologic analyses. Am J Epidemiol. 1986;124(1):17–27. 3521261 10.1093/oxfordjournals.aje.a114366

[6] Pike MC, Bernstein L, Peters RK. Re: “Total energy intake: implications for epidemiologic analyses.”. Am J Epidemiol. 1989;129(6):1312–13. 2729266 10.1093/oxfordjournals.aje.a115254

[7] Howe GR . The first author replies. Am J Epidemiol. 1989;129(6):1314–15.

[8] Kipnis V, Freedman LS, Brown CC, Hartman A, Schatzkin A, Wacholder S. Interpretation of energy adjustment models for nutritional epidemiology. Am J Epidemiol. 1993;137(12):1376–80. 8333419 10.1093/oxfordjournals.aje.a116647

[9] Freedman LS, Kipnis V, Brown CC, Schatzkin A, Wacholder S, Hartman AM. Comments on “Adjustment for total energy intake in epidemiologic studies.”. Am J Clin Nutr. 1997;65(4):1229S–31S. 10.1093/ajcn/65.4.1220S 9094926

[10] Hu FB, Stampfer MJ, Rimm E, Ascherio A, Rosner BA, Spiegelman D, Willett WC. Dietary fat and coronary heart disease: a comparison of approaches for adjusting for total energy intake and modeling repeated dietary measurements. Am J Epidemiol. 1999;149(6):531–40. 10084242 10.1093/oxfordjournals.aje.a009849

[11] Ahmadi-Abhari S, Luben RN, Powell N, Bhaniami A, Chowdhury R, Wareham NJ, Forouhi NG, Khaw K-T. Dietary intake of carbohydrates and risk of type 2 diabetes: the European Prospective Investigation into Cancer-Norfolk study. Br J Nutr. 2014;111(2):342–52. 23880355 10.1017/S0007114513002298

[12] Leite MLC, Prinelli F. A compositional data perspective on studying the associations between macronutrient balances and diseases. Eur J Clin Nutr. 2017;71(12):1365–9. 28853741 10.1038/ejcn.2017.126

[13] Aitchison J . The statistical analysis of compositional data. J R Statist Soc B. 1982;44(2):139–77.

[14] Arnold KF, Berrie L, Tennant PWG, Gilthorpe MS. A causal inference perspective on the analysis of compositional data. Int J Epidemiol. 2020;49(4):1307–13. 32154892 10.1093/ije/dyaa021 PMC7660155

[15] Pearl J, Glymour M, Jewell NP. Causal inference in statistics: a primer. Chichester, United Kingdom: Wiley; 2016.

[16] Geiger D, Verma T, Pearl J. Identifying independence in Bayesian networks. Networks. 1990;20(5):507–34.

[17] Breskin A, Murray EJ. Compositional data call for complex interventions. Int J Epidemiol. 2020;49(4):1314–15. 32529230 10.1093/ije/dyaa084 PMC7660156

[18] Textor J, van der Zander B, Gilthorpe MS, Liskiewicz M, Ellison GTH. Robust causal inference using directed acyclic graphs: the R package ‘dagitty’. Int J Epidemiol. 2016;45(6):1887–94. 28089956 10.1093/ije/dyw341

[19] NatCen Social Research, MRC Elsie Widdowson Laboratory. National Diet and Nutrition Survey Years 1–9, 2008/09–2016/17. 15th ed. [Internet]. Colchester, United Kingdom: UK Data Service; 2019; [Accessed 2021 July 13]. Available from: https://doi.org/10.5255/UKDA-SN-6533-15.

[20] Tu Y-K, Law GR, Ellison GTH, Gilthorpe MS. Ratio index variables or ANCOVA? Fisher's cats revisited. Pharm Stat. 2010;9(1):77–83. 19337988 10.1002/pst.377

[21] Pearson K . Mathematical contributions to the theory of evolution.—On a form of spurious correlation which may arise when indices are used in the measurement of organs. Proc R Soc Lond. 1897;60:489–98.

[22] Ibsen DB, Laursen ASD, Würtz AML, Dahm CC, Rimm EB, Parner ET, Overvad K, Jakobsen MU. Food substitution models for nutritional epidemiology. Am J Clin Nutr. 2021;113(2):294–303. 33300036 10.1093/ajcn/nqaa315

[23] Harrington JM, Dahly DL, Fitzgerald AP, Gilthorpe MS, Perry IJ. Capturing changes in dietary patterns among older adults: a latent class analysis of an ageing Irish cohort. Public Health Nutr. 2014;17(12):2674–86. 24564930 10.1017/S1368980014000111 PMC10282272

[24] Tennant PWG, Murray EJ, Arnold KF, Berrie L, Fox MP, Gadd SC, Harrison WJ, Keeble C, Ranker LR, Textor J et al. Use of directed acyclic graphs (DAGs) to identify confounders in applied health research: review and recommendations. Int J Epidemiol. 2021;50(2):620–32. 33330936 10.1093/ije/dyaa213 PMC8128477

