# Supplementary material for: Adjustment for energy intake in nutritional research: a causal inference perspective
Source: Am J Clin Nutr. 2021 Jul 27;115(1):189–98. doi: 10.1093/ajcn/nqab266 (PMC8755101; doi:10.1093/ajcn/nqab266)
Supplement: nqab266_Supplemental_File [file nqab266_supplemental_file.docx]

**Adjustment for energy intake in nutritional research: a causal inference perspective**

Georgia D Tomova, Kellyn F Arnold, Mark S Gilthorpe, Peter WG Tennant

| Variable | Mean ± SD |
| --- | --- |
| Fasting plasma glucose, *mg/dL* | 80 ± 25 |
|  |  |
| Sugars, *kcal* | 250 ± 125 |
| Carbohydrates, *kcal* | 500 ± 250 |
| Fiber, *kcal* | 100 ± 50 |
| Saturated fat, *kcal* | 275 ± 125 |
| Unsaturated fat, *kcal* | 400 ± 200 |
| Protein, *kcal* | 300 ± 150 |
| Alcohol, *kcal* | 175 ± 50 |
|  |  |
| Total energy intake, *kcal* | 2000 ± 400 |
| Remaining energy intake, *kcal* | 1750 ± 400 |

**Supplementary Table 1**Target mean and standard deviation values of the variables in the simulated data.

## Supplementary Figure 1

Path diagram of the simulated data structure and path coefficients. Energy from sugars was assigned a standardized path coefficient of 0.25, carbohydrates 0.33, fiber -0.02 (because the calorific energy of insoluble fiber is not obtainable), saturated fat 0.175, unsaturated fat 0.24, protein 0.15, and alcohol 0.09.

Fasting plasma glucose

Carbohydrates

Non-milk extrinsic sugars

Fiber

Saturated fat

Unsaturated fat

Protein

Alcohol

Z=0.5

Z=0.25

Z=-0.5

Z=0.25

Z=0.5

5.0kg/100kcal

3.3kg/100kcal

-1.0kg/100kcal

3.5kg/100kcal

3.0kg/100kcal

2.5kg/100kcal

4.5kg/100kcal

Z=0.5

Z=0.25
